# Clinical Assessment of Fatigability in Multiple Sclerosis: A Shift from Perception to Performance

**DOI:** 10.3389/fneur.2016.00194

**Published:** 2016-11-07

**Authors:** Bryant A. Seamon, Michael O. Harris-Love

**Affiliations:** ^1^Human Performance Research Unit, Muscle Morphology, Mechanics and Performance Laboratory, Clinical Research Center, Washington DC Veterans Affairs Medical Center, Washington, DC, USA; ^2^Physical Medicine and Rehabilitation, Washington DC Veterans Affairs Medical Center, Washington, DC, USA; ^3^Department of Exercise and Nutritional Sciences, Milken Institute School of Public Health, The George Washington University, Washington, DC, USA

**Keywords:** multiple sclerosis, fatigability, fatigue, clinical assessment, task performance, rehabilitation

## Introduction

Multiple sclerosis (MS) is a neurodegenerative disorder characterized by an inflammatory autoimmune disease process in the central nervous system (CNS) (1). MS presents with clinical impairments based on location and severity of CNS lesions. Fatigue is the most common reported symptom of people with MS (PwMS) (2), and 80–85% of individuals describe it as the most disabling feature of the disease (3, 4). Decreased quality of life (5), limited physical activity (6, 7), and increased rates of depression and anxiety (8, 9) are associated with higher levels of reported fatigue among PwMS. The specific etiology of fatigue in MS is unknown, and it is likely the product of multiple factors rather than a single cause (1).

There is a need for development of a unified taxonomy to help define what people experience when they report fatigue (10, 11). An early attempt to define fatigue was published from the 1981 CIBA Foundation Symposium in “Human Muscle Fatigue: Physiological Mechanisms” by Edwards (12) as “a failure to maintain the required or expected force.” While Edwards provided a simple and direct operational definition, it failed to convey subjective feelings described by PwMS. Enoka and Stuart (13) expanded Edwards’ definition to include perception, stating that fatigue is “an acute impairment of performance that includes both an increase in the perceived effort necessary to exert a desired force and the eventual inability to produce this force.” This definition features Mosso’s dichotomy and is now a commonly used framework within the realm of fatigue research (13). Within this taxonomy, force decrements are considered distinct from sensations that arise from prolonged muscular activity. However, as investigators began to uncover multiple mechanistic causes for fatigue, they began to label fatigue with descriptors consisting of the independent variables studied. Examples of this trend include cognitive fatigue, peripheral fatigue, and central fatigue among others. Beyond cohesive operational definitions, the limited ability to isolate components of Enoka and Stuart’s expanded definition explains, in part, why so little progress has been made in addressing clinically reported fatigue symptoms (14).

Kluger et al. (10) presented a taxonomy that attempts to reunite the developing silos of fatigue work by returning to Enoka and Stuart’s definition. He calls for the common language of fatigue to be divided into two well-defined categories, distinguishing between the perception of fatigue and fatigability. “Perception of fatigue” defines subjective sensations related to an individual’s symptom complaint and is the result of homeostatic and psychological factors. “Fatigability” relates to task performance and is defined by a change in performance relative to an objective criterion. Enoka and Duchateau (11) presented additional framework for viewing fatigue as a symptom that has a trait characteristic and can be influenced by state variables. This view of fatigue allows researchers to measure the effects of short-term and modifiable state variables on the long-term trait characteristic of fatigue (i.e., the perception of fatigue or fatigability). The approach of Enoka and Duchateau (11) encourages investigators to emphasize their assessment methodology and the task dependency of fatigue, while minimizing use of obtuse modifiers or descriptors that lack clarity and yield little insight into causative factors. Developing and conceptualizing unified operational definitions of fatigue holds ramifications for clinical practice. It is our view that a combination of poorly defined taxonomies, unknown etiology, and vague clinical descriptions have made fatigue difficult to quantify during clinical assessment. Therefore, it is not surprising that current treatments are non-specific and yield unsatisfactory outcomes.

The purpose of this paper is to convey the limitations of current fatigue assessments for evaluating task performance fatigability in rehabilitative settings for PwMS. Additionally, we call for the development of clinical tests which can measure the influence of state variables on the trait characteristic of performance fatigability as it relates to function and quality of life.

## The Limitations of Subjective Assessment in Clinical Rehabilitation Environments

The fatigue severity scale (FSS) and fatigue impact scale (FIS) are questionnaires of self-reported fatigue. Both tests are the current primary clinical outcome measures for objectively measuring fatigue symptoms in MS. For example, Latimer-Cheung et al. (15) examined over 30 studies where at least one of these questionnaires was the primary outcome for measuring the impact of exercise on fatigue. While self-reported fatigue remains an important outcome, the use of the FSS and FIS involves limitations associated with questionnaires regarding regression to the mean and response bias (16). Moreover, exercise-based interventions paradoxically show large changes in functional capacity and independence with only mild to moderate changes in fatigue questionnaire scores after skilled physical therapy including aerobic endurance training and progressive resistance training (17, 18). Scores on both the FSS and FIS correlate with disease severity as determined by the extended disability status scale (EDSS) in PwMS (19). However, the FSS and FIS do not adequately reflect functional status established by clinical outcome measures such as the 6-min walk test (20, 21), gait speed, or temporal and spatial components of gait kinematics (22). Questionnaires may have poor association with indices of whole muscle fatigue derived from isometric muscle testing (18). The utility of self-reported fatigue assessments may be constrained by the confounding of qualitative complaints of fatigue by other MS impairments (23). Steens et al. (24) found fatigability in PwMS explained variance in FSS scores; however, more of the variance was explained when adding depression to the regression. This exemplifies how a qualitative complaint or perception can influence subjective tests and highlights the need for developing objective measures of fatigability. In our opinion, the need for outcome measures to separate fatigability from the perception of fatigue is critical in clinical practice environments such as rehabilitative medicine. Clinically measuring fatigability requires careful consideration to develop appropriate performance tasks and valid outcomes (10). Based on this perspective, emphasis needs to be placed on developing clinical assessments correlated with state variables of fatigability to accurately evaluate the trait characteristic of fatigue’s influence on an individual’s function, when determining independence or recovery in PwMS.

## Enhancing Functional Outcomes with Clinical Fatigability Assessment

Historically, PwMS were discouraged from participating in regular exercise to avoid exacerbating fatigue (25). There has been a paradigm shift in the last decade as exercise programs for PwMS have demonstrated promising improvements in functional performance (26). A growing body of evidence suggests that rehabilitative programs for PwMS may enhance quality of life and contribute to maintaining independence throughout the progression of the disease. Several interventions including a resistance-training component, in particular, may be effective for reducing both perceived fatigue and improving functional status (15, 26). However, there is a lack of consensus on the most efficacious exercise modality and dosage for treating reported fatigue in PwMS. Furthermore, it is the authors’ view that the failure to include fatigability measures as the primary outcome in exercise studies limits the ability to draw firm conclusions regarding the efficacy of an exercise prescription on modifying state variables related to performance fatigability in PwMS. This omission may partially explain the limited adoption of strength training and other modes of exercise as viable treatment options for fatigability in PwMS (26).

**Figure 1:**
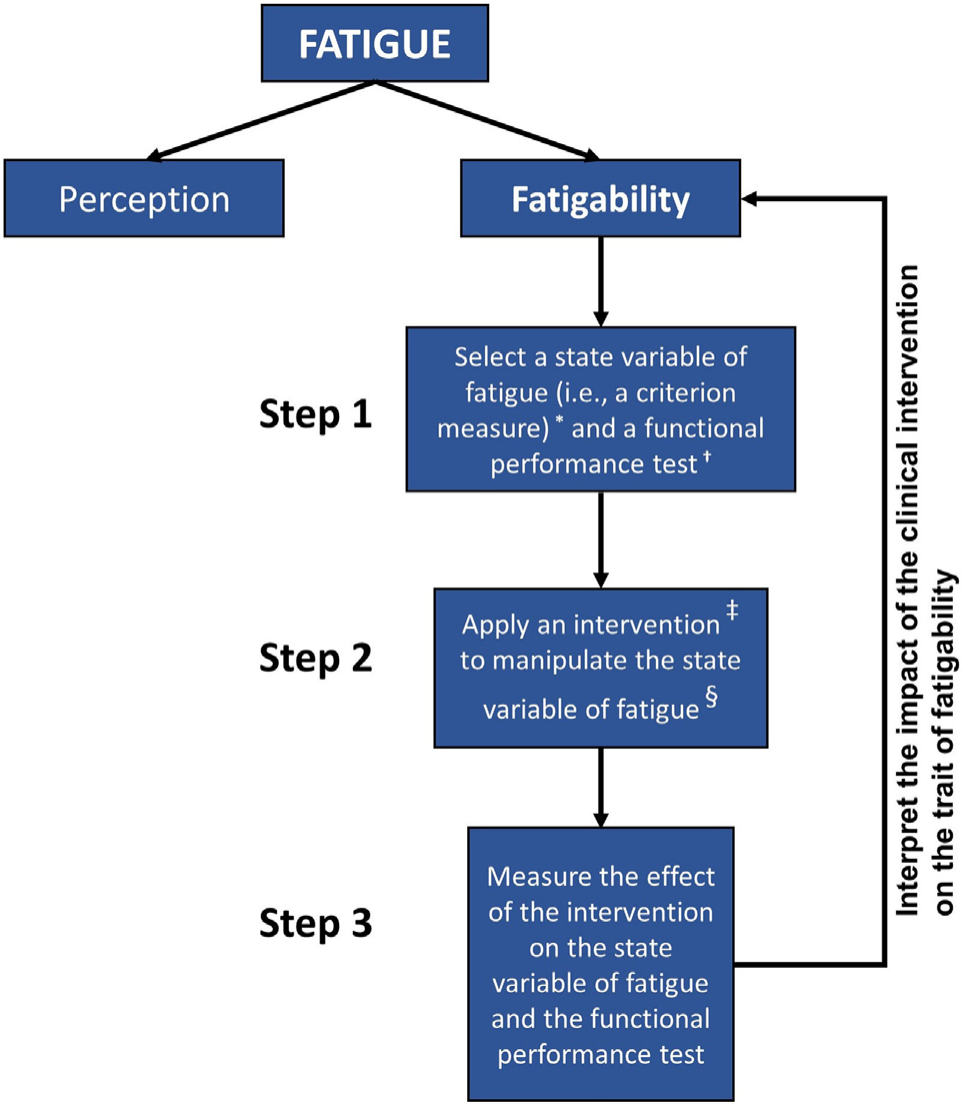
**Hierarchy chart depicting the general steps for associating clinical assessments of fatigability with overall fatigue in individuals with MS**. Self-reported measures of perceived fatigue do not consistently correspond to performance-based measures of fatigability. Valid functional measures of fatigue must be associated with accepted estimates of fatigability and viable for use in rehabilitation settings. Better understanding of the relationship between the performance-based criterion measures and the trait characteristic of fatigability may aid our approach to the assessment and treatment of those people with multiple sclerosis. Notes: *Examples of criterion measures include the duration of task sustainability, rate of change in force production, power, voluntary activation, reaction times, heart rate, mean arterial pressure, core temperature, and outcomes of muscle morphology. ^†^Examples of functional performance tests include the Adult Myopathy Assessment Test (AMAT), Short Physical Performance Battery Protocol (SPPB), and Dalgas’ Functional Capacity Test (FCT). ^‡^Examples of state variables include the exercise prescription, modality of exercise, and exercise environment. ^§^Examples of intervention to manipulate a state variable: a progressive resistance exercise program or an aerobic endurance exercise program.

Reframing how investigators characterize fatigue in MS may guide subsequent research efforts within this area of inquiry. Enoka and Duchateau (11) have proposed three levels of analysis for measuring the impact of fatigability on human performance. They propose first selecting a criterion measure of performance modulated by fatigue and then identifying a laboratory test that measures the performance of the criterion measure. Finally, they suggest conducting studies to determine the significance of adjustments to the modulating factors limiting performance on the laboratory test (where the modulating factors are state variables, and the reported symptom of fatigue relates to the trait characteristic of the individual). In Figure 1, the authors present a similar strategy that can be adopted for clinical measurement as well.

In a rehabilitative setting, clinical assessments of fatigability should be associated with functional tasks of daily living. Steens et al. (24, 27) have begun to look at state characteristics of fatigability, including muscular strength and capacity (both criterion measures of performance), which are associated with trait levels of fatigue reported in PwMS. While it is helpful to understand that state variables of fatigability relate to the overall trait of fatigue, we still have limited knowledge about how these variables are associated with clinical assessment measures. For example, Steens’ observations did not include limb muscles that are more likely to mimic functional task requirements and mobility in MS. Little is known about the relationship between state variables of MS-related fatigue and the performance of limb musculature involved in tasks such as walking, which is heavily evaluated as a functional outcome related to independence in this population.

Our stance is that the development of clinical assessments, which manipulate state variables under fatiguing tasks, is the most direct way to evaluate the trait characteristic of fatigue on functional performance. Of key importance is designing tests which can best mimic functional requirements of daily living. Current functional exams of performance in MS are not strongly associated with fatigue as a trait characteristic (20–22). Tests which may hold promise include the Short Physical Performance Battery Protocol (28), Adult Myopathy Assessment Tool (29), and the Functional Capacity Test developed by Dalgas et al. (30). These tests attempt to combine various repetitive tasks which mimic daily function. However, it is our opinion that the validity of these tests would be enhanced by better understanding their association with trait characteristics of fatigue. The construct validity of the aforementioned tests would be strengthened by determining the relationship between the scores obtained from these functional assessments and performance values from various fatigue tests in both aerobic and anaerobic conditions. This approach would better characterize how state variables such as metabolic requirements or muscle actions would align with task specificity measured within a clinic. Dalgas et al. (31) state that limited existing literature on fatigability responses in aerobic compared to anaerobic environments has made assessment of both endurance and strength exercise trials difficult. Another outcome often overlooked when evaluating state variables of muscle performance is anaerobic recovery. Our view is that further inquiry into anaerobic recovery relative to muscle capacity could provide meaningful insights into how fatigability affects temporal aspects of functional task performance. Such information could help practitioners adjust exercise prescription and monitor training adaptations.

Assessment of muscle morphology as a state variable may also provide valuable insights concerning performance fatigability. Previous work by Kent-Braun et al. (32) and Wens et al. (33) highlight changes in muscle related to cross-sectional area, size, composition, and fiber type in PwMS. Importantly, diminished muscle cross-sectional area and greater levels of intramuscular adipose tissue are associated with poor performance with repeated or sustained functional tasks (34). While methods to characterize skeletal muscle are often invasive or difficult to implement in rehabilitation settings (32, 35), alternative approaches involving ultrasound have shown that proxy measures of muscle tissue composition are also associated with impaired performance (36, 37). Further study regarding muscle morphology using clinically viable methods may advance our understanding of the state variables of fatigability in MS.

Further evaluation of the impact of modulating state variables on fatigability in clinical rehabilitation settings will be challenging. The current trend of measuring multiple state variables of fatigue at once has masked the clinically significant changes that can occur through interventions (15). However, building a consensus regarding a unified fatigue taxonomy, and the further development of standardized methods to assess state variables of fatigability, will advance the larger goal of implementing effective rehabilitation treatment for MS-related fatigue.

## Conclusion

Fatigue is a vague symptom that defies simple characterization. Due to this ambiguity, there is a need for quantifiable clinical measures of performance fatigability as they relate to the rehabilitation of PwMS. Assessing fatigability may be critical for understanding the relationship between an individual’s function and reported fatigue symptoms. Performance-based fatigability testing provides an in-depth view of muscle function as a state variable related to activities of daily living and mobility. With focused clinical testing, rehabilitation professionals can track the response to interventions and make recommendations on specific exercise prescriptions for PwMS. Taking these steps will help clinicians guide PwMS toward the goals of minimizing debilitating fatigue, improving functional performance, and enhancing their quality of life.

## Author Contributions

BS and MH-L were responsible for the conceptual idea; performed the literature review; prepared the figure; drafted the manuscript; edited and revised the manuscript critically for important intellectual content; and approved the final draft.

## Conflict of Interest Statement

The authors declare that the research was conducted in the absence of any commercial or financial relationships that could be construed as a potential conflict of interest.
